# P2Y_12_ Receptors in Tumorigenesis and Metastasis

**DOI:** 10.3389/fphar.2018.00066

**Published:** 2018-02-02

**Authors:** Patrizia Ballerini, Melania Dovizio, Annalisa Bruno, Stefania Tacconelli, Paola Patrignani

**Affiliations:** ^1^Department of Psychological, Health and Territorial Sciences, Università degli Studi “G. d’Annunzio” Chieti-Pescara, Chieti, Italy; ^2^Center for Aging and Translational Medicine, Università degli Studi “G. d’Annunzio” Chieti-Pescara, Chieti, Italy; ^3^Department of Neuroscience, Imaging and Clinical Science, Center for Aging and Translational Medicine, Università degli Studi “G. d’Annunzio” Chieti-Pescara, Chieti, Italy

**Keywords:** P2Y_12_, ADP, platelets, cancer, metastasis

## Abstract

Platelets, beyond their role in hemostasis and thrombosis, may sustain tumorigenesis and metastasis. These effects may occur via direct interaction of platelets with cancer and stromal cells and by the release of several platelet products. Platelets and tumor cells release several bioactive molecules among which a great amount of adenosine triphosphate (ATP) and adenosine diphosphate (ADP). ADP is also formed extracellularly from ATP breakdown by the ecto-nucleoside-triphosphate-diphosphohydrolases. Under ATP and ADP stimulation the purinergic P2Y_1_ receptor (R) initiates platelet activation followed by the ADP-P2Y_12_R-mediated amplification. P2Y_12_R stimulation amplifies also platelet response to several platelet agonists and to flow conditions, acting as a key positive feed-forward signal in intensifying platelet responses. P2Y_12_R represents a potential target for an anticancer therapy due to its involvement in platelet-cancer cell crosstalk. Thus, P2Y_12_R antagonists, including clopidogrel, ticagrelor, and prasugrel, might represent potential anti-cancer agents, in addition to their role as effective antithrombotic drugs. However, further studies, in experimental animals and patients, are required before the recommendation of the use of P2Y_12_R antagonists in cancer prevention and progression can be made.

## Introduction

The number of cancer cases, which are diagnosed each year continues to rise, primarily due to an aging population. According to a recent report by Weir et al. (2015), this increase, in the United States, in all races and all sites, will be of 24,1% among men and of 20.6% among women within 2020. Cancer, along with cardiovascular disease (CVD), remains the most common cause of death. Most of the cancer-related deaths are due to the metastatic process, which is regulated by different mechanisms including the interaction of cancer cells with other cellular components present either in the tumor microenvironment or in the bloodstream (De Palma et al., 2017). Emerging evidence has recognized a central role for platelets in both tumor progression and metastasis (Contursi et al., 2017) and paraneoplastic thrombocytosis is observed in more than 30% of subjects diagnosed with different types of solid tumors, where it is associated to poor prognosis (Haemmerle et al., 2017).

Interestingly, it has been pointed out that low-dose aspirin (75–100 mg), which mainly targets platelets, reduces the incidence and mortality of colorectal cancer (CRC) and other types of solid tumors (Rothwell et al., 2010, 2011, 2012; Patrignani and Patrono, 2016).

In updating its recommendations, the US Preventive Services Task Force stated that low-dose aspirin should be used for the primary prevention of CVD and CRC in adults aged 50–59 years “who have a 10% or greater 10-year CVD risk, are not at increased risk for bleeding, have a life expectancy of at least 10 years, and are willing to take low-dose aspirin daily for at least 10 years”(Bibbins-Domingo and U.S. Preventive Services Task Force, 2016).

Collectively, the results of clinical and experimental studies support the notion that targeting platelet activation is a promising strategy for cancer prevention.

The antiplatelet effect of low-dose aspirin is dependent on the preferential inhibition of platelet cyclooxygenase (COX)-1 which translates into a virtually complete inhibition of thromboxane (TX) A_2_, i.e., a potent lipid mediator which acts as an amplifier of the response to primary platelet agonists, such as thrombin and collagen.

**FIGURE 1:**
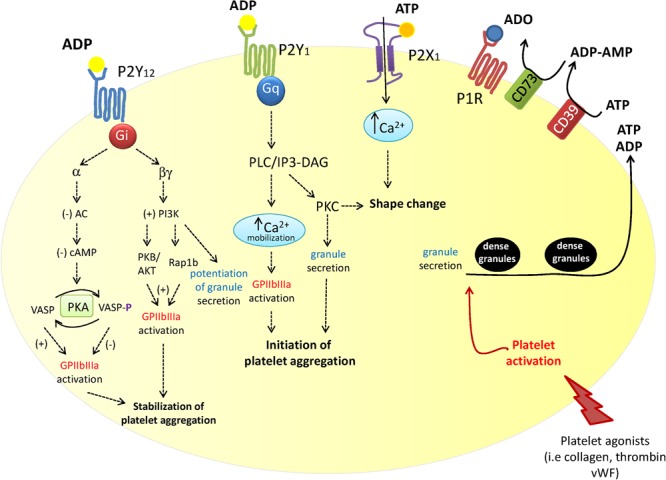
Schematic overview of the contribution of the three P2 purinergic receptor subtypes, P2Y_1_, P2Y_12_, and P2X_1_ in platelet activation. P2Y_12_ receptor plays a central role in ADP-mediated platelet activation. Platelet exposure to primary platelet agonists including thrombin, collagen and Von Willebrand factor (vWF) results in their adhesion followed by the release of ATP and ADP from dense granules. Extracellular ATP is rapidly metabolized into ADP, AMP and finally to adenosine by the ecto-enzymes CD39 and CD73. Secreted ATP and ADP, as well as ADP deriving from ATP degradation, activate P2Y_1_, P2Y_12_, and P2X_1_ receptors (as depicted). P2Y_12_ receptor stimulation modulates the growth and stability of thrombus by potentiating platelet dense granule release, platelet aggregation and procoagulant activity (Kunapuli et al., 2003).

Another secondary agonist for platelets is adenosine diphosphate (ADP) which is released from platelet dense granules in response to the primary agonists (McNicol and Israels, 1999) (**Figure 1**). Adenine nucleotides and nucleosides [adenosine triphosphate (ATP), ADP and adenosine] modulate platelet aggregation, shape change and the release of alpha granules (Enjyoji et al., 1999; Burnstock, 2017). The role of ATP, ADP, and adenosine in platelet function is controlled by an organized enzymatic chain, including ecto-nucleoside triphosphate diphosphohydrolase (CD39) and ecto-5′-nucleotidase (CD73), which catalyzes the hydrolysis of released ATP into ADP, adenosine monophosphate (AMP) and finally to adenosine (Bakker et al., 1994; Robson et al., 2006; Burnstock, 2017). Extracellular nucleotides act on platelets through distinct receptors belonging to the P2 ATP family: the P2Y_1_ and P2Y_12_ metabotropic, G protein-coupled receptors, involved in transient platelet shape change and platelet aggregation (Burnstock, 1972) and the P2X_1_ ionotropic receptor (Burnstock, 1972; Cattaneo et al., 2002; Oury et al., 2004; Gachet, 2012) (**Figure 1**).

The expression and function of P2Y_12_ in other cell types is still poorly investigated. P2Y_12_ congenital deficiency results in bleeding disorders characterized by a platelet impaired response to ADP (Cattaneo et al., 1992; Nurden et al., 1995; Cattaneo, 2011), but, in these patients, information is lacking on potential modifications induced in other tissues and organs.

In this minireview, the expression pattern in both normal and malignant cells and the signaling pathways of the P2Y_12_ receptor (P2Y_12_R) will be overviewed. The P2Y_12_R involvement in cancer development, progression, and metastasis, as well as the role of P2Y_12_R antagonists in these pathological processes, will be also discussed.

## P2Y_12_ Receptor Expression and Function in Normal and Malignant Cells

A full platelet aggregation in response to ADP occurs by the stimulation of P2Y_1_ receptor, followed by P2Y_12_ activation (**Figure 1**). P2Y_1_ is a Gq-coupled receptor that initiates ADP-induced platelet aggregation through the stimulation of phospholipase C and phosphatidylinositol-signaling pathway. P2Y_12_R is a seven transmembrane domain receptor. It mediates the inhibition of adenylate cyclase and, in turn, cyclic AMP (cAMP) production via the coupling to Gα_i_ leading to impaired protein kinase A (PKA) activation and a subsequent inhibition of vasodilator-stimulated phosphoprotein (VASP), which restrains either secretory or adhesive events in platelets (**Figure 1**).

Vasodilator-stimulated phosphoprotein phosphorylation flow cytometry assay is used to monitor platelet responsiveness to P2Y_12_ targeted antiplatelet therapy (particularly in tailoring the treatment with the oral P2Y_12_ inhibitor clopidogrel) (Gachet, 2012; Fitzgerald and FitzGerald, 2013; Siller-Matula et al., 2013; Danese et al., 2016).

P2Y_12_R activation also recruits Gβγ subunits, causing phosphoinositide-3-kinase-(PI3K) dependent Akt phosphorylation and Rap1b activation, a key positive regulator pathway for the integrin GPIIb/IIIa. In this way, the sustained activation of P2Y_12_R contributes to thrombus stabilization. PI3Kβ isoform has been reported to be essential for ADP-induced TXA_2_ generation and platelet aggregation (Garcia et al., 2010) and to cooperate with PI3Kγ isoform in sustaining integrin activation (Cosemans et al., 2006; Schoenwaelder et al., 2007).

P2Y_12_R-Gi signaling leads positive regulation of other intracellular pathways including extracellular-signal-regulated kinase (ERK), myosin light chain kinase and Src family kinases as well as to membrane lipid shifts toward a pro-coagulant state such as phosphatidylserine and P-selectin exposure (Leon et al., 2003; Gachet, 2012). By acting on P2Y_12_R, ADP also contributes to the release of several agonists such as TXA_2_ (Cattaneo, 2015).

P2Y_12_R was originally found to be expressed only by platelets (Hollopeter et al., 2001), however further studies reported that it is functionally present in microglial cells, the resident immune cells of the brain, where it can play a role in their activation (Haynes et al., 2006). *In vivo* experiments confirm a role for P2Y_12_ in microglia. P2Y_12_-deficient mice showed a diminished early response to focal injury and microglia from these animals was much less responsive to purine nucleotides in terms of cell migration (Haynes et al., 2006). Recently, it was shown that ADP stimulation of microglia P2Y_12_R induced ERK1/2 and paxillin Ser83 phosphorylation, which play a role in the regulation of focal adhesions and actin cytoskeleton rearrangement (Lee et al., 2012). Moreover, in hippocampal slices, the receptor has been shown to stimulate process extension through the activation of integrin- extracellular matrix interaction (Ohsawa et al., 2010; Swiatkowski et al., 2016).

P2Y_12_R has also been shown to regulate migration of vascular smooth muscle cells (VSMCs). In these cells, ADP, through P2Y_12_-Gα_i_ activation, inhibited cAMP/PKA signaling pathway resulting in cofilin dephosphorylation, actin disassembly and, as a consequence, an increase in VSMCs motility and migration (Niu et al., 2017).

A role for P2Y_12_R in inflammation and immune modulation has been recently reported (Wang et al., 2004; Diehl et al., 2010; Burnstock and Boeynaems, 2014; Cattaneo, 2015; Hechler and Gachet, 2015). Interestingly, it has been shown that platelets negatively affect the adoptive T cell therapy (ACT) in cancer by producing high levels of active TGFβ. Moreover, platelets are the only cell type known so far to constitutively express the TGFβ-docking receptor glycoprotein A repetitions predominant (GARP) which allows them to capture TGFβ from both other cells and the extracellular matrix. This platelet-specific TGFβ-GARP-axis seems to play a critical role by constraining the antitumor activity of T cell immunity (Rachidi et al., 2017). In B16-F1 melanoma-C57BL/6 mice, clopidogrel, a P2Y_12_R antagonist, in combination with aspirin made the ACT therapy highly effective compared to the control group, which received water. Indeed, most mice survived without relapse for more than 3 months (Rachidi et al., 2017).

The P2Y_12_R expression has been also recently reported in human eosinophils; in these cells, it caused the release of eosinophil peroxidase (Muniz et al., 2015).

Expression of P2Y_12_R in cancer cells has been poorly investigated. The receptor protein has been found in glioma and astrocytoma cells (Jin et al., 2001; Czajkowski et al., 2002; Burnstock and Di Virgilio, 2013) where it has been reported to increase cancer cell proliferation. In basal condition, C6 glioma cells expressed predominantly P2Y_1_ mRNA with lower levels of P2Y_12_ mRNA, but, when the cells were cultured in serum-free medium, the expression of P2Y_1_ mRNA decreased, whereas that of P2Y_12_ significantly increased (Czajkowski et al., 2004). In these conditions, ADP enhanced ERK1/2 phosphorylation and PI3K signaling by activating the P2Y_12_R (Czajkowski et al., 2004).

More recently, P2Y_12_ expression has been also described in breast cancer cell lines (Sarangi et al., 2013). The baseline expression of the receptor protein was low in both normal breast epithelium (MCF 10A cells) and in human breast cancer cell lines, namely MCF7 and MDA-MB-231 (Sarangi et al., 2013). Interestingly, as for the glioma cells, the P2Y_12_ protein levels were enhanced by serum starvation. Also, cell treatment with cisplatin, a well-known chemotherapeutic agent, enhanced P2Y_12_ expression in breast cancer cells (Sarangi et al., 2013; Dasari and Tchounwou, 2014). The inhibition of P2Y_12_ reduced cisplatin-mediated increase of hypoxia-inducible factor 1-alpha, a factor involved in the resistance to cytotoxic therapy (Ai et al., 2016; Zhao et al., 2016), in angiogenesis and in metastatic processes (Choi et al., 2016; Wang et al., 2016).

In platelet rich plasma from healthy subjects the P2Y_12_R antagonist cangrelor reduced the production of ADP-stimulated vascular endothelial growth factor (VEGF) (Bambace et al., 2010) a key protein in angiogenesis. Moreover, platelet secretion of other proangiogenic molecules, including IL-1α, IL-1β, GM- CSF, MMP-1 and uPAR, can be controlled by P2Y_12_R inhibitors in non-small cell lung cancer cell-stimulated platelets (Wu et al., 2015).

These evidence, together with the increasing interest in the anticancer properties of metal-based compounds (Chen et al., 2017), have recently lead to the development of innovative gold (III) complexes of prasugrel, a newer oral P2Y_12_R inhibitor, with promising chemotherapeutic activities (Benkli, 2016).

Finally, a role for P2Y_12_R has been postulated in neuropathic pain, which is often caused by chronic diseases such as cancer. It has been shown that peripheral nerve injury was able to increase the expression of different ATP P2Y receptors, including P2Y_12_, in microglial cells present in the spinal dorsal horn (Kobayashi et al., 2008, 2012). More recently, in a rat model of tongue cancer pain, obtained by inoculation of squamous cell carcinoma cells, a marked activation of microglia through P2Y_12_R was found in the trigeminal spinal subnucleus caudalis. This resulted to be associated with increased excitability of nociceptive neurons and consequent allodynia after mechanical stimulation. The administration of the P2Y_12_ antagonist MRS2395 strongly reduced the “nocifensive” behavior and microglial activation in these animals (Tamagawa et al., 2016).

## P2Y_12_R Antagonists and Cancer

**FIGURE 2:**
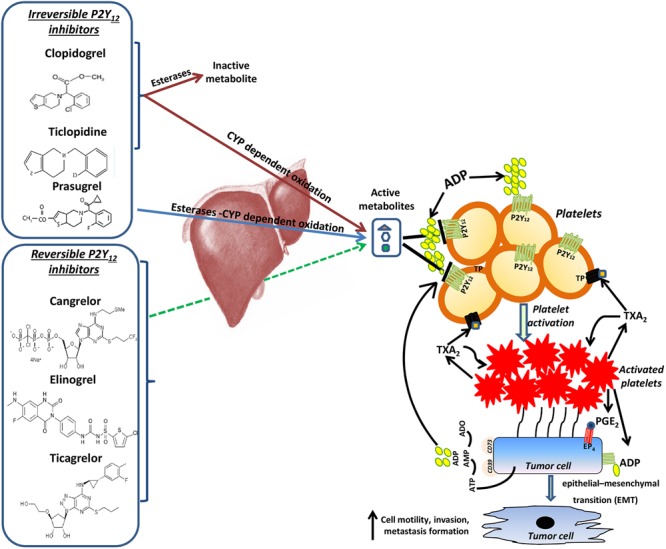
The potential contribution of P2Y_12_ receptor in cancer and metastasis. P2Y_12_ receptor (R) antagonists include thienopyridines, such as ticlopidine, clopidogrel and prasugrel, that irreversibly inhibit P2Y_12_R upon metabolic conversion into active metabolites by the hepatic cytochrome P-450 system (Cattaneo, 2010; Schrör et al., 2012) and ticagrelor, cangrelor and elinogrel, which reversibly and directly bind the receptor without any need for bioactivation (Cattaneo, 2010; Schrör et al., 2012). By targeting P2Y_12_R these drugs may contribute to constrain metastasis development through the prevention of platelet activation by ADP. Once activated, platelet-derived PGE_2_ and a direct platelet-tumor cell interaction synergize to promote epithelial-mesenchymal transition (EMT), migration and metastasis formation (Guillem-Llobat et al., 2016). Moreover, like platelets, cancer cells have been shown to release a significant amount of ATP (Beigi et al., 1999; Pellegatti et al., 2008; Burnstock and Di Virgilio, 2013), which is hydrolyzed into ADP through the activity of the ecto-enzymes CD39 (Bakker et al., 1994; Robson et al., 2006; Burnstock, 2017). Extracellular ADP, deriving from both activated platelets and cancer cells, in turn could activate P2Y_12_R expressed both in platelets and in malignant cells, thus triggering a vicious circle, which could contribute to cancer progression and dissemination.

At present, there are two groups of P2Y_12_ antagonists (**Figure 2**) which, after aspirin, are the most widely prescribed antiplatelet agents in CVD. Thienopyridines, including ticlopidine, clopidogrel, and prasugrel, irreversibly inhibit P2Y_12_R upon metabolic conversion into active metabolites by the hepatic cytochrome P-450 system (Cattaneo, 2010; Schrör et al., 2012). On the other hand, ticagrelor, cangrelor, and elinogrel reversibly and directly bind the receptor without any need for bioactivation (Cattaneo, 2010; Schrör et al., 2012) (**Figure 2**).

Kohga et al. (1981) firstly demonstrated that ticlopidine treatment suppressed the formation of pulmonary nodules in rodents injected with a B16 melanoma or AH130 rat ascites hepatoma cells. In a rodent model of spontaneous pulmonary metastasis, ticlopidine was able to inhibit lung metastasis when administered *per os* (Kohga et al., 1981).

More recently, in a mouse model of either spontaneous or experimentally induced lung metastasis, obtained by injection of Lewis lung carcinoma (LLC) cells and B16 melanoma cells respectively, P2Y_12_ deficiency reduced the weight of lung metastasis without affecting the primary tumors (Wang et al., 2013). This suggests a role for the ADP receptor in promoting the metastatic process. This hypothesis was strengthened by the observation that platelets from P2Y_12_-deficient mice significantly reduced the invasiveness of LLC cells. Moreover, P2Y_12_ deficiency reduced the capability of the cancer cells to stimulate the production of active TGFβ1 from platelets. This, in turn, resulted in the prevention of platelet-induced epithelial-mesenchymal transition (EMT) of the tumor cells (Wang et al., 2013), a process known to contribute pathologically to cancer progression and metastasis.

In agreement with these data, Guillem-Llobat et al. (2016) recently reported that the interaction of platelets with HT29 human colon carcinoma cells leads to the induction of EMT in tumor cells associated to enhanced cell mobility. When the cells were co-cultured with platelets in the presence of ticagrelor, both the down-regulation of E-cadherin, an epithelial cell marker, and the enhanced migratory capacity of HT29 cells were inhibited (Guillem-Llobat et al., 2016). Ticagrelor also caused the simultaneous inhibition of TXB_2_ (the stable hydrolysis product of TXA_2_) and of prostaglandin (PG)E_2_ production, suggesting an inhibitory effect on the release of arachidonic acid from platelet membrane phospholipids (Guillem-Llobat et al., 2016).

Ovarian cancer has been shown to be a potential target for P2Y_12_ inhibitors. Ticagrelor, given by daily gavage, reduced the growth of primary tumors in rodent models of ovarian cancer (Cho et al., 2017). The drug caused a significant decrease in Ki67 immunostaining (a proliferation marker) in tumors resected form ticagrelor treated mice compared to controls. A direct role of P2Y_12_ in ovarian cancer cells was ruled out since Western blot analysis did not detect the receptor at the protein levels, and knocking down P2Y_12_ by siRNA or CRISPR-Cas9 techniques did not affect the cancer cell proliferation either *in vitro* or *in vivo* (Cho et al., 2017).

P2Y_12_ seems to mediate also bone loss under pathological conditions including cancer. In a mouse model of bone metastasis, clopidogrel increased bone mineral density and trabecular bone volume compared to controls (Su et al., 2012). Accordingly, in a mouse model of tumor-associated bone loss, the P2Y_12_ deficiency protected the animals from trabecular bone loss. Moreover, in the P2Y_12_^-/-^ mice, the number and surface of osteoclasts significantly increased in the tumor-bearing wild-type animals, was similar to the controls (Su et al., 2012).

Combination of antiplatelet agents has been poorly evaluated. In a mouse model of chronic immune-mediated hepatitis B that progresses to hepatocellular carcinoma, the treatment with low-dose aspirin and clopidogrel caused a marked reduction in the development of hepatomas. Also, the overall mass of the hepatomas resulted to be lower in mice treated with the combination therapy. At the time when 75% of vehicle-treated mice were found dead, only 20% of the animals treated with aspirin plus clopidogrel had died. Notably, the combined antiplatelet treatment did not cause significant bleeding side effects in these animals (Sitia et al., 2012).

Although the few published preclinical studies suggest a potential role for P2Y_12_ antagonists in chemoprevention and/or in potentiating the effect of cytotoxic drugs (Sarangi et al., 2013; Dasari and Tchounwou, 2014; Pandey et al., 2014) there are no results from randomized clinical trials (RCTs) aimed to assess the effects of these drugs on cancer and metastasis.

Concerns have been raised on the possible association between P2Y_12_ antiplatelet therapy and solid tumor growth or metastatic dissemination, even if the evidence for this harmful association was not sufficient to modify the clinical practice (Serebruany et al., 2015). A recent population-based cohort study has been published comparing the association of cancer risk between treatment with aspirin alone and aspirin in combination with clopidogrel. At least 3 years of follow-up was guaranteed by the study and patients with a diagnosis of cancer within the first year were excluded. Newly diagnosed cases of cancer, with the exception of melanoma skin tumor, were 21.977 out of 184.781 subjects, the primary endpoint being the time until first diagnosis (Leader et al., 2017). Breast, colorectal, prostate, and lung cancer were the most common cancer types. The study showed that there was not a higher risk for cancer in subjects assuming the combined treatment compared to aspirin used alone (HR 0.92, 95% CI: 0.86–0.97) and suggested that clopidogrel could even reduce cancer incidence (Leader et al., 2017). Similarly, an FDA meta-analysis, carried out to assess the effects of clopidogrel on death rates from all causes, showed that the dual antiplatelet therapy with aspirin and clopidogrel, given for 12 months or longer, was safe. Indeed, there was no increased risk for cancer-related deaths compared to aspirin and clopidogrel administered for 6 months or less, or to aspirin alone (FDA, 2015).

A systematic review and meta-analysis were performed with the aim to verify whether thienopyridines increased cancer mortality and cancer events. Nine studies have been analyzed including six RCTs and three retrospective cohort studies for a total number of 282,084 participants. All the studies reported on clopidogrel, whereas only two specifically reported on prasugrel. The exposure to clopidogrel showed no association with increased odds of cancer (OR 0.70, 95% CI: 0.66–0.75, *n* = 1). Furthermore, no significant difference in cancer event rate was pointed out for prasugrel when compared to clopidogrel (OR 1.10, 95% CI: 0.89–1.37, *n* = 2). Similar results were obtained on the analysis of cancer mortality. On the whole, these data do not support concerns for a class effect of thienopyridines in increasing the cancer event rate and/or mortality (Kotronias et al., 2017).

## Conclusion and Perspectives

Several lines of evidence show that the cross-talk of cancer cells with stromal cells (such as immune cells and fibroblasts) and platelet-derived products induce a novel phenotype, which allows them to invade the healthy tissue around, to enter the bloodstream and to colonize distant tissues. The role of platelets in conditioning tumor microenvironment, by releasing several bioactive mediators and microvesicles, is increasingly recognized. Platelets and cancer cells have been shown to release a significant amount of ATP and ADP (Beigi et al., 1999; Pellegatti et al., 2008; Burnstock and Di Virgilio, 2013).

Given the site-specific nature of cancer, along with the specific mechanisms that different cancer cells can develop to activate platelets and *viceversa* the final ATP/ADP effect will depend on the P2 receptor subtypes expressed and activated (Burnstock and Di Virgilio, 2013). Thus, an improved knowledge of the signaling induced by the activation of P2Y_12_R during the interaction of platelets with cancer cells is needed. Interestingly, the PLATelet inhibition and patient Outcomes (PLATO) study showed that ticagrelor exerts an anti-inflammatory effect higher than clopidogrel (Thomas and Storey, 2015). Ticagrelor can inhibit the adenosine transporter ENT1 (Cattaneo et al., 2014). This increases the extracellular concentration of the nucleoside, which is a recognized modulator of inflammatory and immune responses. Thus, clinical research, focused on targeted biomarkers, is needed to clarify the role of P2Y_12_R-antagonists in these mechanisms, which are relevant in cancer development and metastasis.

Moreover, an in-depth characterization of P2Y_12_R expression in tumor cells is necessary to enlighten potential novel direct therapeutic activities of P2Y_12_ antagonists. On the other hand, further findings on P2Y_12_R activity in non-malignant cells should be useful, since they could help monitoring the possible adverse effects caused by the systemic administration of P2Y_12_-targeted drugs.

In conclusion, although emerging evidence suggests a rationale for targeting P2Y_12_R to constrain tumor progression, metastasis development, and pain and to potentiate the responses to conventional therapies, a definitive evidence of the anticancer effect of P2Y_12_R antagonists is lacking. Further studies of basic and clinical research are urgently needed in this setting to put a final word on the role of the purinergic signaling in cancer development and metastasis.

## Author Contributions

Conception of work: PB and PP; design of work: PB and PP; drafting of manuscript: PB, MD, ST, and AB; critical revision of manuscript: PB and PP; final approval of work: PB, MD, ST, AB, and PP; agreement to be accountable for all aspects of the work in ensuring that questions related to the accuracy or integrity of any part of the work are appropriately investigated and resolved: PB, MD, ST, AB, and PP.

## Conflict of Interest Statement

The authors declare that the research was conducted in the absence of any commercial or financial relationships that could be construed as a potential conflict of interest. The reviewers ED, MM and handling Editor declared their shared affiliation.
